# A New Perspective for Potential Organ Damage Due to Iron-Mediated Oxidation in Thalassemia Major Patients

**DOI:** 10.3390/jcm12062422

**Published:** 2023-03-21

**Authors:** Funda Eren, Ayça Koca Yozgat, Esra Firat Oğuz, Salim Neşelioğlu, Rıdvan Firat, Dilek Gürlek Gökçebay, Hüsniye Neşe Yarali, Namık Yaşar Özbek, Özcan Erel

**Affiliations:** 1 Department of Medical Biochemistry Laboratory, Ankara Bilkent City Hospital, 06800 Ankara, Turkey; 2 Department of Pediatric Hematology and Oncology, Ankara Bilkent City Hospital, 06800 Ankara, Turkey; 3 Department of Medical Biochemistry, Yıldırım Beyazıt University, 06010 Ankara, Turkey

**Keywords:** antioxidant, oxidant, thalassemia, transfusion

## Abstract

Background: The aim of this study is to develop new perspectives to prevent or reduce potential organ damage due to iron-mediated oxidation in thalassemia major patients. Methods: Seventy patients were included in this study. Blood samples were taken from the patients before and after transfusion. Total thiol, native thiol, disulfide, disulfide/native thiol percentage ratio, ischemia modified albumin (IMA), total antioxidant status (TAS), total oxidant status (TOS), and ferroxidase levels were determined. Additionally, undepleted thiol level (UTL) was determined as a new parameter associated with organ damage. Results: After transfusion, the levels of native thiol, total thiol, disulfide, TAS, ferroxidase, and TOS were higher, while the IMA levels and disulfide/native thiol percent ratio were lower. Significant correlations were found between antioxidant and oxidant tests before and after transfusion. Additionally, a negative correlation was found between the TOS and UTL levels of the patients measured before the transfusion. Conclusion: In the present study, transfusion therapy increased both oxidation and the antioxidant levels. In addition, the term UTL has been introduced as a parameter that enables the determination of the oxidation level that may cause potential organ damage in transfusion-dependent thalassemia patients.

## 1. Introduction

Thalassemias are hereditary blood disorders due to anomalies in the synthesis of the chains of hemoglobin [1]. ß-thalassemia (β-thalassemia) is a condition characterized by microcytic hypochromic anemia, an abnormal peripheral blood smear with reticulocytes, and decreased hemoglobin ß chain synthesis resulting in low amounts of hemoglobin A (HbA) [2]. Thalassemia is one of the most common inherited hemoglobinopathies and the prevalence is approximately 2% in Turkey [3].

Red cell suspension transfusion is the mainstay of therapy in patients with major thalassemia. A regular transfusion schedule and chelation therapy aimed at reducing iron overload allow for normal growth and development and can improve overall prognosis [1,4]. Intracellular enzymes such as superoxide dismutase, catalase, glutathione peroxidase, and some other components such as vitamin E and reduced glutathione (thiol groups) provide protection from oxidative stress [5]. Overdose oxidative stress on cells can cause the oxidation of proteins, lipids, and DNA, leading to cell death and organ damage. Oxidative stress is believed to exacerbate the symptoms of many diseases, including hemolytic anemias. There are several studies showing oxidative stress in the pathogenesis of hemoglobinopathies such as ß-thalassemia, glucose-6-phosphate dehydrogenase deficiency, hereditary spherocytosis, and congenital dyserythropoietic anemias [6,7,8].

Irreversible organ damage occurs in many organs as a result of oxidative reactions occurring due to excess iron in patients with major thalassemia [9]. Although iron accumulation is tried to be prevented by chelation therapy, the main problem is organ damage caused by oxidation. It is very important to determine quantitatively and evaluate the oxidation level together with chelation therapy in transfusion-dependent thalassemia patients.

The parameters selected in this study were chosen according to their ability to evaluate the presence of antioxidants and oxidants in the serum both cumulatively and in detail.

Thiol groups are organic compounds that are at the forefront of antioxidant molecules and contain a sulfhydryl (-SH) group in their structure. It is suggested that oxidative stress leads to macromolecular damage and disruption of thiol redox circuits leading to abnormal cell signaling and dysfunctional redox control [2,10]. Most of the plasma thiol pool is derived from proteins, and some from molecules such as low molecular weight cysteine, cysteinylglycine, glutathione, homocysteine, and gama glutamylcysteine. When thiol groups in serum are oxidized, they form disulfide bonds. Disulfide bonds are converted back to thiol groups by the action of antioxidants in the serum. This condition is reversible and is in balance under physiological conditions. However, in many diseases, this balance is disturbed [11]. Thiol–disulfide balance tests consist of native thiol, total thiol, disulfide, and disulfide/native thiol ratio parameters. Total thiol is expressed as the sum of disulfide and native thiol levels, while disulfide/native thiol percent ratio is expressed as the ratio of oxidant and antioxidant levels [10].

Undepleted thiol level (UTL) is a term used for the first time for thalassemia patients. UTL is the mathematical difference of serum native thiol values measured after and before transfusion (UTL = serum native thiol value measured after transfusion–serum native thiol value measured before transfusion). The difference arises depending on the oxidation state of the patient prior to transfusion. It suggests that UTL may be related to the elimination of oxidation products found in thalassemia patients by the antioxidant thiol groups in the erythrocyte suspension.

Ischemia modified albumin (IMA) is a marker that has been used in the diagnosis of ischemic disease such as coronary artery disease in recent years. In the presence of ischemia, when the balance between the oxidant and antioxidant system is disturbed, albumin undergoes structural and functional modifications. Thus, the metal-binding ability of albumin is impaired and the level of IMA increases [2]. Total antioxidant status measurement test (TAS) and total oxidant status measurement test (TOS) provide cumulative information about antioxidant and oxidant status in the serum [12,13]. Ferroxidase, a copper-containing oxidase protein, has oxidase activities against polyamines, polyphenols, and inorganic iron ions. It is also critical in iron metabolism. The primary physiological role of ferroxidase is associated with redox reactions in the organism [14,15]. Catalyzing the oxidation of ferrous ions (Fe^2+^) to the less reactive ferric state (Fe^3+^), the form required for iron binding to apotransferrin, is associated with the antioxidant activity of ceruloplasmin. Ferrous iron ions are known to be an important catalysts of hydroxyl radical formation. Ferroxidase activity removes reactive iron ions from the plasma, thus preventing the stimulation of lipid peroxidation by iron ions [16].

Up to date, there are several studies about the relationship between oxidative stress and ß-thalassemia. To our knowledge, this is the first comprehensive study aiming to evaluate the oxidant and antioxidant status before and after transfusion by detecting serum total thiol, native thiol, disulfide, IMA, TAS, TOS, and ferroxidase levels. The aim of this study is to develop new perspectives to prevent or reduce organ damage due to iron-mediated oxidation in thalassemia major patients.

## 2. Materials and Methods

This study was conducted at the pediatric hematology department and medical biochemistry laboratory of the hospital. Seventy patients who were diagnosed with ß-thalassemia major receiving regularly scheduled blood transfusions were included in this study. Transfusion treatments are started when the patients are 6–9 months old and are performed regularly once a month. All patients are given deferasirox (30–40 mg/kg/day) as an iron chelator. In our country, all blood components are manufactured and distributed by the Turkish Red Crescent. According to the transfusion program, the patients were transfused with ABO and subgroup compatible, pre-storage leukofiltered, and non-irradiated packed erythrocyte units in a volume of 15 cc/kg every 3–4 weeks. The RBC units were preserved with SAG-M (saline, adenine, glucose, mannitol) and stored for a maximum 7 days. Patients included those with the diagnosis of thalassemia intermedia or minor, endocrinological disorders, cardiovascular and infectious diseases, and cancer. Additionally, insufficient samples were excluded from our study. Informed consents were obtained from the parents of all subjects. Two blood specimens were collected from the patients before and 2 h after transfusion into serum gel separator tubes and centrifuged at 1500× *g* for 20 min. Sera were aliquoted and stored at −80 °C until the analysis. Total thiol and native thiol were measured with a novel automated spectrophotometric method. Disulfide and disulfide/native thiol percentage ratios are calculated using the measured parameters [10]. UTL is the mathematical difference of serum native thiol values measured after and before transfusion (UTL = serum native thiol value measured after transfusion–serum native thiol value measured before transfusion). IMA level was measured spectrophotometrically by the method based on the cobalt binding capacity principle of albumin defined by Bar-Or et al. [17]. Total antioxidant status (TAS) and total oxidant status (TOS) levels were measured also with an automated direct measurement method developed by Erel O [13,18]. For ferroxidase enzyme activity, ferric iron ions (Fe^3+^) were measured kinetically in a spectrophotometer using acetohydroxamic acid [19].

Our study was approved by Ankara City Hospital Ethics Committee number E2-22-1437.

The distribution of the data was evaluated by using Kolmogorov–Smirnov analysis. Descriptive analyses were expressed as mean ± standard deviation for normally distributed variables and as median (interquartile range) for non-normally distributed variables. Paired *t*-test was used to determine the significance level of the difference between groups for normally distributed data, and the Wilcoxon test was used for data that were non-normally distributed. In addition, Pearson correlation analysis was performed for normally distributed variables and Spearman correlation analysis was performed for non-normal distribution. Categorical variables were presented as numbers and percentages. All statistical calculations were made using SPSS (Statistical Package for Social Sciences) software (v.22; IBM, Armonk, NY, USA). *p* values less than 0.05 were considered statistically significant for all analyses. Figures were created using GraphPad Prism (Version 6.0; GraphPad Software Inc., La Jolla, CA, USA).

## 3. Results

The study included 70 patients with the diagnosis of ß-thalassemia receiving regular transfusion therapy, 36 (51.4%) females and 34 (48.6%) males. The mean age of the patients was 8.83 ± 4.32 years.

**Figure 1:**
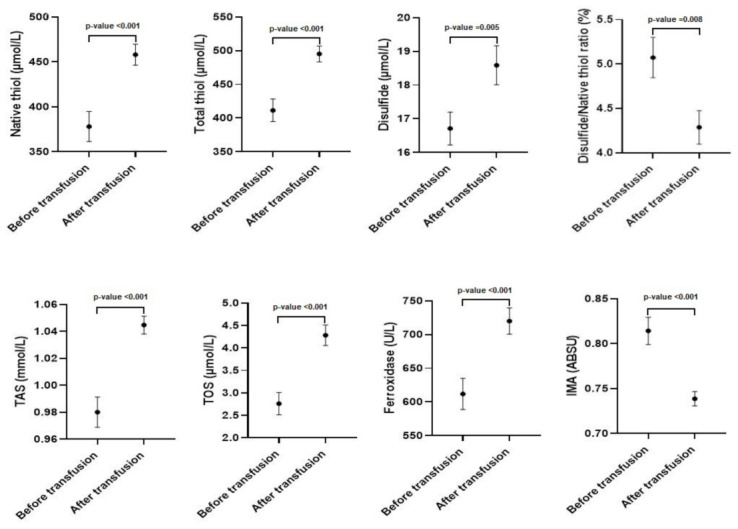
Antioxidant and oxidant parameters before and after transfusion in thalassemia patients ($\bar{x}$ ± $\sigma_{\bar{x}}$) (*n* = 70).

After transfusion native thiol, total thiol, disulfide, TAS, ferroxidase, and TOS levels were found to be significantly higher (*p* < 0.001). IMA level and disulfide/native thiol percent ratio were found to be significantly lower after transfusion (*p* < 0.001) (Table 1 and Figure 1).

Significant correlations were found between antioxidant and oxidant tests before and after transfusion (Table 2 and Table 3).

There is a significant negative correlation between the TOS levels and the UTL levels of the patients measured before the transfusion (r = −0.436; *p* < 0.001).

## 4. Discussion

Blood transfusions, ineffective erythropoiesis, and intravascular hemolysis result in iron overload in many body tissues of ß-thalassemia patients. Iron overload leads to increased ROS (reactive oxygen species) production and oxidative stress [20]. Overproduction of ROS can cause cellular toxicity and organ dysfunctions [21]. Antioxidants protect cells against oxidative stress damage by eliminating or preventing ROS production [22]. It has been hypothesized that changes in erythrocytes in ß-thalassemia are associated with a constant oxidative stress resulting from excessive intracellular precipitation of alpha-globin chains and release of free iron [23,24]. Iron overload in ß-thalassemia leads to an enhanced generation of ROS and results in oxidative stress in the body. For compensation, antioxidants in the body take role to protect erythrocytes from oxidative stress. In previous studies, it has been indicated that increased antioxidant status was probably due to the increase in oxidative stress in ß-thalassemia patients receiving blood transfusions and iron chelators [4,25].

In our study, we evaluated the oxidant and antioxidant status of ß-thalassemia patients before and after blood transfusion. According to our findings, the levels of the antioxidants (TAS, native and total thiol, and ferroxidase) were significantly higher and oppositely the levels of the oxidants (disulfide/native thiol ratio and IMA) were significantly lower after transfusion. Moreover, the TOS levels, which show the oxidative status, were significantly higher after transfusion. The antioxidant parameters showed statistically significant correlation with each other and also the oxidant parameters showed statistically significant correlation with each other. Moreover, antioxidant and oxidant parameters were negatively correlated. In the study, strong negative correlations were obtained between the levels of native thiol, TAS, and ferroxidase, which are important antioxidant parameters, and IMA levels, which is one of the oxidative stress indicators. These results show that the oxidant state is dominant in patients with ß-thalassemia before transfusion. These results indicate that the antioxidant–oxidant balance is impaired in patients with ß-thalassemia before transfusion. It was observed that the disulfide/native thiol ratio, which indicates the antioxidant–oxidant balance through thiol metabolism, was also impaired before transfusion. In addition, a significant positive correlation between disulfide/native thiol and IMA levels supports the existence of oxidative stress. Additionally, significant positive correlation values observed between the antioxidant parameters native thiol, TAS, and ferroxidase levels are important for the reliability of the measurements (Table 2 and Table 3). It is known that oxidative stress increases in ß-thalassemia patients [26]. It is thought that the reason for the increase in post-transfusion antioxidant status in our patients receiving regular transfusion therapy is related to the erythrocyte suspension given.

TAS shows the antioxidant status of the body [12]. According to some studies, total antioxidant status (TAS) was not different between ß-thalassemia patients and healthy controls [27]. In our study, TAS levels were significantly higher after transfusion comparing with before transfusion. This result indicates that the transfusion therapy contributes to the increased antioxidant status.

TOS levels were also significantly elevated after transfusion in the present study. The elevated iron levels in ß-thalassemia patients, even after transfusion, may lead to the elevation of lipid peroxides and oxidants through Fenton reaction [27].

In previous studies, serum IMA levels of ß-thalassemia patients were found to be significantly higher than controls [15,28], considering the presence of oxidative stress, ROS production, hypoxia, and anemia in ß-thalassemia [29,30] elevated IMA levels are expected to be observed in these patients. In our study, we found high IMA levels before transfusion in ß-thalassemia patients, consistent with the literature. After transfusion, the IMA levels were significantly lower. Transfusion causes an increase in hemoglobin levels and a decrease in hypoxia and oxidative stress. The presence of lower IMA levels in patients after transfusion may be associated with these changes.

Ceruloplasmin ferroxidase activity converts iron from the ferrous to the ferric form that prevents the formation of ROS and reduces oxidative damage through the Fenton reaction [15,31]. In a previous study, ferroxidase activity in thalassemia patients was found to be higher as compared to healthy controls [14]. The high ferroxidase activity observed in ß-thalassemia may be attributed to mechanisms related to iron overload. In our study, ferroxidase levels were significantly elevated after transfusion. Transfusion therapy increases the iron overload, resulting in increased ferroxidase levels to prevent the formation of ROS via the Fenton reaction by oxidation of Fe^2+^ to Fe^3+^.

Thiol and disulfide homeostasis plays an important role in the antioxidant protection and detoxification process [2]. Our study was carried out to examine the changes in thiol and disulfide homeostasis. Total and native thiol levels were found to be significantly higher after transfusion. Disulfide levels were also found to be higher after transfusion. Disulfide bounds are the products of the oxidation of the thiols. The increase in disulfide bounds is due to the increase in thiol groups after transfusion. The decrease in the ratio of disulfide/native thiol indicates the decreased oxidative stress associated with thiol metabolism after transfusion. In addition, considering the metal chelation feature of thiol groups [32], it is thought that increased thiol levels after transfusion may increase the effectiveness of treatment by contributing to iron chelation therapy as well as antioxidant activity [33].

In patients with transfusion-dependent thalassemia, erythrocyte transfusion is the most important step in the treatment. However, as a result of oxidative reactions that occur due to excessive iron accumulation resulting from regular blood transfusion, irreversible damage may occur in many organs [9]. In this study, the increase in TOS levels after transfusion indicates the presence of iron-mediated oxidative stress in line with the literature. In addition, it was observed that TAS and native thiol levels, which reflect the antioxidant status, increased after transfusion. Studies to date have only mentioned the increase in oxidative stress and ignored the increase in antioxidants that eliminates some of the oxidative stress and thus partially reduces organ damage. To our knowledge, this point of view has been reported for the first time in this study.

In addition, we think that determining thiol levels in serum samples taken before and after transfusion and keeping the UTL value under control can reduce organ damage. One of the causes of organ damage in patients with thalassemia major is damage to macromolecules due to iron-mediated oxidation and resulting in cell death. Although iron accumulation is tried to be prevented by chelation therapy, the main problem is cell damage caused by oxidation. For this reason, quantitative determination of oxidation level together with chelation therapy in transfusion-dependent thalassemia patients would be beneficial. In this study, native thiol levels, which have antioxidant properties, were measured in serum samples taken from patients before and after transfusion. Native thiol levels increased after transfusion in thalassemia patients (Table 1, Figure 1). The difference depends on the oxidation state of the patient prior to transfusion. In the present study, the difference in thalassemia patients was named as the thiol level that remained undepleted (undepleted thiol level—UTL). It was shown that there is a significant negative correlation between UTL and serum TOS levels measured before transfusion (r = −0.436; *p* < 0.001). In other words, the patients with low oxidation levels before transfusion have high UTL values, or the patients with high oxidation levels before transfusion have low UTL values. Based on this, it can be predicted that keeping the UTL values of patients under control with thiol-containing drugs (e.g., alpha lipoic acid, n-acetyl cysteine) may help to limit oxidative damage that may cause cell damage. There are many studies showing that the use of alpha-lipoic acid in thalassemia major patients reduces cardiovascular risk factors [34], limits damage caused by iron-mediated oxidative stress [35], and has an excellent iron chelating property [33]. These positive effects of alpha-lipoic acid suggest that it may be a way to determine the damage observed in thalassemia patients by UTL measurement. The quantitative determination of oxidation by UTL measurement is a prediction that cell damage known to occur due to oxidation can be detected.

One of the limitations of our study was the limited number of patients. Additionally, we could evaluate only a limited number of oxidant and antioxidant parameters. The evaluation could be performed at the time of the diagnosis of the patients and the first transfusion therapy. Moreover, more detailed evaluation of the patients with organ damage is needed Additionally, any parameter showing the cell damage directly could not be evaluated.

In conclusion, transfusion therapy in major thalassemia patients not only increased the oxidation levels but also increased the antioxidant levels. In addition, the term “undepleted thiol level” has been introduced as a parameter that enables the determination of the oxidation levels that may cause cell damage in transfusion-dependent thalassemia patients. Thus, it was suggested that the UTL value could be presented as a quantitative value related to the prediction of cell damage. According to the data, we believe that the results may contribute to the effectiveness of transfusion used in the treatment of ß-thalassemia and to limit possible organ damage.

## Figures and Tables

**Table 1:** The values of antioxidant and oxidant parameters before and after transfusion in thalassemia patients.

Parameters	Before Transfusion	After Transfusion	*p*-Values
Native thiol (µmol/L)	378.0 ± 150.2	458.0 ± 105.6	<0.001 *
Total thiol (µmol/L)	411.4 ± 152.1	495.2 ± 107.7	<0.001 *
Disulfide (µmol/L)	16.7 ± 3.4	18.5 ± 4.2	=0.005 *
Disulfide/Native thiol ratio (%)	5.1 ± 1.1	4.3 ± 0.7	=0.008 *
TAS (mmol/L)	0.98 ± 0.05	1.04 ± 0.06	<0.001 *
Ferroxidase (U/L)	611.8 ± 208.1	720.1 ± 176.9	<0.001 *
IMA (ABSU)	0.76 (0.14)	0.73 (0.06)	<0.001 **
TOS (µmol/L)	2.14 (0.89)	3.7 (2.15)	<0.001 **

TAS—total antioxidant status; TOS—total oxidant status; IMA—ischemia modified albumin; ABSU—absorbance unit; indicates a significant statistical difference with *p* < 0.05; * used paired sample *t*-test; ** used Wilcoxon signed rank test.

**Table 2:** The correlation analysis of oxidant and antioxidant status before transfusion.

Parameters		Ferroxidase	TAS	TOS	IMA
Native thiol	r	0.704	0.492	0.438	−0.775
	*p*	<0.001 *	<0.001 *	<0.001 *	<0.001 *
Total thiol	r	0.710	0.505	0.438	−0.785
	*p*	<0.001 *	<0.001 *	<0.001 *	<0.001 *
Disulfide	r	0.256	0.328	0.099	−0.331
	*p*	0.02 *	0.003 *	0.377	0.002 *
Disulfide/Native thiol	r	−0.567	−0.376	−0.298	0.692
	*p*	<0.001 *	<0.001 *	0.007 *	<0.001 *
Ferroxidase	r	1	0.479	0.378	−0.659
	*p*		<0.001 *	<0.001 *	<0.001 *
TAS	r	0.479	1	0.186	−0.703
	*p*	<0.001 *		0.094	<0.001 *
TOS	r	0.378	0.186	1	−0.291
	*p*	<0.001 *	0.094		0.008 *
IMA	r	−0.659	−0.703	−0.291	1
	*p*	<0.001 *	<0.001 *	0.008 *	

* indicates a significant statistical difference with *p* < 0.05. r: correlation coefficient.

**Table 3:** The correlation analysis of oxidant and antioxidant status after transfusion.

Parameters		Ferroxidase	TAS	TOS	IMA
Native thiol	r	0.421	0.164	0.300	−0.679
	*p*	<0.001 *	0.140	0.006 *	<0.001 *
Total thiol	r	0.419	0.174	0.319	−0.677
	*p*	<0.001 *	0.118	0.003 *	<0.001 *
Disulfide	r	0.089	0.133	0.252	−0.132
	*p*	0.429	0.235	0.022 *	0.238
Disulfide/Native thiol	r	−0.286	−0.139	0.007	0.547
	*p*	0.009 *	0214	0.951	<0.001 *
Ferroxidase	r	1	0.106	0.260	−0.361
	*p*		0.341	0.018 *	0.001 *
TAS	r	0.106	1	0.185	−0.140
	*p*	0.341		0.096	0.209
TOS	r	0.260	0.185	1	−0.158
	*p*	0.018 *	0.096		0.155
IMA	r	−0.361	−0.140	−0.158	1
	*p*	0.001 *	0.209	0.155	

* indicates a significant statistical difference with *p* < 0.05. r: correlation coefficient.

## Data Availability

Data can be made accessible upon demand.
